# Case of Extensive Injury by Machinery, to the Upper Extremity

**Published:** 1857-07

**Authors:** Thos. Hall

**Affiliations:** Toulon, Illinois


ARTICLE III.—*Case of extensive Injury, by Machinery, to the Upper Extremity.* By THOS. HALL, M. D., of Toulon, Illinois.

*August* 10*th*, 1855.—Called in consultation, after night, to see a young man, aged 20, who, two hours before, had been caught in the wheels of a threshing machine. Found him pale and faint, with a pulse rapid and scarcely perceptible. He had been caught about the upper third of the right forearm between cog-wheels, and that portion of the arm was completely destroyed. The arm then escaped to the upper part of the humerus; from that point to the axilla every muscle, blood-vessel, nerve, etc., was destroyed; nothing was left but the deltoid, and that was somewhat injured both on its anterior and posterior portions. The lower portion of the shoulder joint was ground off. The first and second ribs, immediately below the axilla, were partly ground through, and the third rib was bare, but not slenuded. Not a vestige of the contents of the axilla could be discovered. No pulsation could be felt in the subclavian artery, or, if so, so near to its origin as to be uncertain. I proposed, with the concurrence of the attending physician, to amputate at the shoulder-joint; but any operative procedure was firmly forbid by the young man's father. All necessary directions were given, and I left him for the night.

*August* 11*th*, 8 *o'clock A. M.*—His general condition somewhat improved. There had been no hemorrhage of importance during the night. But unless we would guarantee his recovery, his father still persisted in our letting him alone. It was now evident that the destruction of the soft parts covering the upper portion of the chest was added to the general mass torn away by the machinery, and I felt somewhat thankful that the obstinacy of the father prevented me from forming a loose flap that could not possibly unite to any living portion of muscle. Desperate as the case appeared to be, I ventured, against the opinion of two other physicians, to give a favorable prognosis, having once seen, not at the time, but during its progress, a case of the same character, only a little more serious. At this visit I told the father, that when he consented to let his physicians control the treatment, if sent for, that I would come, but not till then. The next morning I was sent for, and amputated the humerus about two inches below its head, saving all the deltoid that appeared to have sustained no injury. There was no hemorrhage, and the young man felt better immediately afterward—asked for some pickled cucumber, which he was permitted to have, and the same evening was carried on a litter three miles to his father's house, expressing himself as feeling better for his ride. My health at the time was miserable. I did not see him again for six days, when everything was going on favorably. The wound healed by granulation, and he is now in good health. I have been concise, but I hope not too much so, in my description of this case. My motive in laying it before the society is to encourage my young brethren to give their patients the salutary influence of hope in every case that is not necessarily fatal, and to caution them against probing or disturbing a wound when no information can be gained by the procedure that can benefit the patient; but, on the contrary, fatal consequences might supervene from the disturbance.

